# Supplementary material for: Discovery of Epipodophyllotoxin-Derived B2 as Promising XooFtsZ Inhibitor for Controlling Bacterial Cell Division: Structure-Based Virtual Screening, Synthesis, and SAR Study
Source: Int J Mol Sci. 2022 Aug 14;23(16):9119. doi: 10.3390/ijms23169119 (PMC9408963; doi:10.3390/ijms23169119)
Supplement: Supplementary file 1 [file ijms-23-09119-s001.zip › ijms-1846795-supplementary.pdf]

Discovery of Epipodophyllotoxin-Derived B_2 as Promising *Xoo*FtsZ Inhibitor for Controlling Bacterial Cell Division: Structure-Based Virtual Screening, Synthesis and SAR Study

Ying-Lian Song, Shuai-Shuai Liu, Jie Yang, Jiao Xie, Xiang Zhou*, Zhi-Bing Wu, Li-Wei Liu, Pei-Yi Wang, and Song Yang*

State Key Laboratory Breeding Base of Green Pesticide and Agricultural Bioengineering, Key Laboratory of Green Pesticide and Agricultural Bioengineering, Ministry of Education, Center for R&D of Fine Chemicals of Guizhou University, Guiyang, 550025, China.

* Corresponding author.

E-mail: jhzx.msm@gmail.com (YS), zhoux1534@163.com (ZX).

1. *In vitro* Antibacterial Bioassay (Turbidimeter Test)

In this study, all the synthesized target compounds were evaluated for their antibacterial activities against *Xoo* by the turbidimeter test *in vitro*.[1-3] Dimethyl sulfoxide (DMSO) in sterile distilled water was treated as a blank control, bismerthiazol and thiodiazole copper was treated as positive controls, simultaneously. 40 μL of NB medium (1.0 g of yeast powder, 3.0 g of beef extract, 5.0 g of peptone, 10.0 g of glucose and 1000 mL of distilled water; pH = 7.0−7.2) containing *Xoo*, which was incubated on the phase of logarithmic growth, was added to 5 mL of solvent NB containing test compounds and positive control at different concentrations, the concentrations were chosen in two times declining trend to make sure that the EC_{50} values are inside the concentration ranges tested). The inoculated test tubes were incubated at 28 ± 1 °C and continuously shaken at 180 rpm for 36−48 h until the *Xoo* were incubated on the logarithmic growth phase. The growth of the medium was monitored by a multi-mode microplate reader (Cytation™5 multi-mode readers and Synergy™ H1 multi-mode readers, BioTek Instruments, Inc. USA), which was measuring the optical density at 595 nm (OD_{595}) given by turbidity-corrected values = $OD_{\text{bacterial wilt}} - OD_{\text{no bacterial wilt}}$, and the inhibition rate I was calculated by $I = (C - T)/C \times 100\%$. C is the corrected turbidity values of bacterial growth on untreated NB (blank control), and T is the corrected turbidity values of bacterial growth on treated NB. By using the SPSS 17.0 software and the obtained inhibition rates at different concentrations, a regression equation was provided. The results of antibacterial activities (expressed by EC_{50}) against *Xoo* were calculated from the equation, and the value was within the concentration ranges. The experiment was repeated three times.

2. *In vivo* antibacterial activity test

The curative and protective activity in potted plants of compound against rice bacterial leaf blight were determined by Schaad's method with some slight modifications.[4-6] Thiodiazole copper (20% suspending agent), the bactericides registered for rice bacterial leaf blight, and served as the positive control samples. The curative activity of compound was determined by potted plants via reducing rice

bacterial leaf blight under controlled conditions in a greenhouse. After sowed the rice seeds of "Fengyouxiangzhan" about 7 weeks, rice leaves were inoculated with *Xoo*, which was incubated at logarithmic growth using sterilized scissors by scissors-dipped method. One day after inoculation, 200 mg L^{-1} compound solution was uniformally sprayed onto the rice leaves until dripping down, whereas distilled water, which contain same percent DMSO was uniformally sprayed onto the rice leaves, served as the negative control plants. Then, all inoculated rice plants were placed in a plant growth chamber (28 °C and 95% RH). At 14 days after sprayed, the disease index of the inoculated rice leaves was measured. Similarly, the protection activity in potted plants for reducing rice bacterial leaf blight of compound was also conducted under controlled conditions in a greenhouse. After sowed the rice seeds of "Fengyouxiangzhan" about 7 weeks, 200 mg L^{-1} compound solution was uniformally sprayed onto the rice leaves until dripping down, whereas distilled water was uniformally sprayed onto the negative control plants. One day after spraying, *Xoo*, which was incubated at logarithmic growth, was inoculated on the rice leaves using sterilized scissors. The grown champer was employed to inoculated rice plants (28 °C and 95% RH). At 14 days after inoculation, the disease index of the inoculated rice leaves was measured. The control efficiencies I (%) for the curative and protection activities are calculated by the following equation. In the equation, C is the disease index of the negative control and T is the disease index of the treatment group. control efficient I (%) = (C-T)/C ×100. Statistical analysis was conducted by ANOVA with software SPSS 20.0. Different uppercase letters following the control efficiency values indicate that there is significant difference ($P < 0.05$) among different treatment group.

3. Determination of minimum inhibitory concentration (MIC)

In this section, the minimum inhibitory concentration (MIC) values of all target compounds against *Xoo* were tested by using the NCCLS method.[7-8] *Xoo* cells were incubated in fresh NB medium and grew at OD_{595} = 0.6. Then, 200 μL of inoculum (the suspension with an OD_{595} = 0.6 was mixed with fresh NB medium at 1:1000

(v/v)) was inoculated onto the 96-well microplates. After that, target compounds were dissolved in dimethyl sulfoxide (DMSO), and an equivalent DMSO was treated as a blank control. The concentrations were chosen in two times declining trend to make sure that the MIC values are inside the concentration ranges tested. Finally, the prepared 96-well microplates were incubated in a constant temperature incubator at 28 °C for 48 h. The lowest concentrations of the tested compounds, which did not show any visual growth after OD_{595} evaluation, were determined as MIC.

4. Purification of the recombinant *Xoo*FtsZ

The sequence of *Xoo*FtsZ gene was obtained from NCBI database. The forward primer *Nde*I was (5'-GGCCCCAAGGGGTTATGCTAGT-3') and the reverse primer *Hind*III was (5'-GATCCCGCGAAATTAATACG-3'). The PCR fragment was digested with *Nde*I and *Hind*III and ligated into the vector pET30(+) cleaved with the same enzymes. To obtain the *Xoo*FtsZ, BL21Gold(DE3) pLysS strain cells transformed with pET30(+) were incubated at 37 °C in LB medium until OD_{595} = 0.6. Then, these cells were induced by supplementing 0.5 mM IPTG and subsequently incubated for another 14 h at 16 °C. The cells were harvested by centrifugation (3000 ×g, 10 min, 4°C) and then were sonicated in two kinds of ice-cold binding buffers (20 mM phosphate (pH 7.4), 500 mM NaCl, 30 mM imidazole, 1 mM EDTA and 1 mM dithiothreitol for the fluorescent titration experiment; 50 mM HEPES-KOH (pH 7.4), 500 mM NaCl, 30 mM imidazole, 1 mM EDTA and 1 mM dithiothreitol for the GTPase activity test). After that, the supernatant was gathered by centrifugation (11000×g, 10 min, 4 °C). 10His-*Xoo*FtsZ was purified by a Ni-NTA column and eluted with a linear gradient of 30–600 mM imidazole. The protein solution was collected, and desalted by desalting column (5 × 5 mL HiTrap Desalting column, GE Healthcare, USA). The final protein concentration was determined by using a protein determination kit (BCA kit; Boster Biological Technology Co., Ltd., China) according to the protocol of the manufacturer.

5. FtsZ assembly affected by target compounds via TEM

The experimental method and detailed steps of this experiment referred to previous report.[9] *Xoo*FtsZ (20 μM) was incubated without or with different concentrations (0 μM, 100 μM and 200 μM) of $\mathbf{A_7}$ in 50 mM HEPES-KOH buffer (pH 8.0) containing

50 mM KCl, 1.25 mM GTP and 2.5 mM $MgCl_2$ for 5 min at 28°C. The formed FtsZ aggregations were transferred to Formvar-carbon-coated copper grids, negatively stained with 1% phosphotungstic acid and observed under a transmission electron microscope.

6. Measurement GTPase activity for Primary Screening

The GTPase activity was performed by measurement of the amount of Pi released during the assembly of *Xoo*FtsZ using a standard Malachite Green assay kit (Cayman chemical, USA) and referred to previous reported methods.[8,10-11] Briefly, 1 μM *Xoo*FtsZ was incubated without or with different concentrations of compounds in 50 mM HEPES-KOH buffer (pH 8.0) containing 50 mM KCl and 1 mM EDTA on ice for 10 min. Subsequently, 2.5 mM $MgCl_2$ and 1.25 mM GTP were added and the reaction mixture was incubated at 28 °C for 20 min. Then, 50 μL samples were quenched by the addition of 5 μL acid solution at 25 °C for 10 min. Then, 15 μL malachite green solution was added to the samples and incubated at 25 °C for 20 min in the dark. Finally, the amount of Pi release was measured the absorption at 620 nm and recorded by Cytation™5 multi-mode readers (BioTek Instruments, Inc. USA).

7. The cytotoxicity of NRK-52E cells affected by compounds

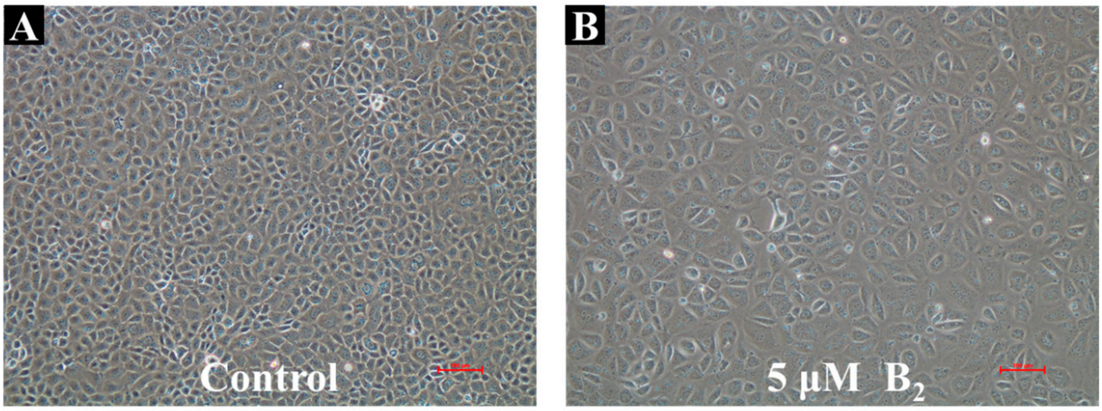

Figure S1. The cytotoxicity of NRK-52E cells affected by compounds DMEP and B_2 at 5 μM.

8.1 General synthetic procedure for Synthesis of compound A_1.

The DMAP (0.30 g, 749.29 μmol) and potassium hydroxide (0.30 g, 749.29 μmol)

were added into a solution of 5 mL *N*, *N'*-dimethylformamide, and the mixture was reacted at room temperature for 10 min. After that, epoxybromopropane was supplemented in the mixture. After the reaction progress was completed, the crude products were extracted three times with 30 mL ethyl acetate, and the organic phase was then further washed with saturated aqueous solution of ammonium chloride and water. Moreover, the separated organic layer was removed under vacuum. Finally, target compound **A_1** was further purified by solvent-extraction together with thin thin-layer chromatography (CH_2Cl_2 : CH_3OH = 100:1, V : V).

8.2 General synthetic procedure for Synthesis of compound B_1-B_3

A mixture of DMEP (100.00 mg, 249.76 μmol), DMAP (30.51 mg, 249.76 μmol) and EDCI (47.88 mg, 249.76 μmol) was dissolved in 5 mL *N*, *N'*-dimethylformamide. Subsequently, the reaction was reacted at room temperature for 12 h. Moreover, 2-ethylbutanoic acid (29.01mg, 249.76 μmol) was supplemented in the mixture. After the reaction progress was completed, the crude products were extracted three times with 15 mL ethyl acetate. The organic phase was then further washed with saturated aqueous solution of ammonium chloride and water, and dried by sodium sulfate. Finally, after the removal of the solvent under vacuum, the compound **B_1** was obtained and further purified by solvent-extraction together with thin-layer chromatography (CH_2Cl_2 : CH_3OH = 100:1, V : V).

9. ^{1}H NMR, ^{13}C NMR and HRMS spectra of target compounds **A_1**, **B_1-B_3**

(5*R*,5a*R*,8a*R*,9*S*)-5-(3,5-dimethoxy-4-(oxiran-2-ylmethoxy)phenyl)-9-hydroxy-5, 5a,8a,9-tetrahydrofuro[3',4':6,7] naphtho[2,3-*d*][1,3]dioxol-6(8*H*)-one (A_1)

A white solid. ^{1}H NMR (400 MHz, DMSO-d_6) δ 6.98 (s, 1H, H-5), 6.75 (s, 1H, H-8), 6.48 (s, 2H, H-2′, 6′), 5.99 (d, J = 4.0 Hz, 2H, OCH_2O), 5.92(d, J = 2.4 Hz, 1H, H-4), 5.63 (d, J = 4.0 Hz, 1H, H-1), 4.52-4.50 (m, 1H, H-8′) , 4.31-4.26 (m, 3H, H-11 + H-7′), 4.13 (dd, J = 3.6, 9.6 Hz, 1H, H-9′), 4.07 (dd, J = 2.8, 11.6 Hz, 1H, H-9′), 3.78-3.74 (m, 1H, H-11), 3.71 (s, 6H, 3′, 5′-OCH_3), 3.22 (dd, J = 4.9, 6.8 Hz, 1H, H-2), 3.24-3.20 (m, 1H, H-3). ^{13}C NMR (101 MHz, DMSO-d_6) δ 179.35, 153.17, 146.73, 146.67, 137.76, 135.50, 133.22, 130.96, 109.50, 106.41, 105.44, 101.29, 74.38, 67.94, 65.85, 56.49, 56.41, 50.79, 45.38, 43.75, 43.22. HRMS (ESI) calcd for $C_{24}H_{24}NaO_9$ ([M+Na]$^+$), 479.1318; found, 479.1313.

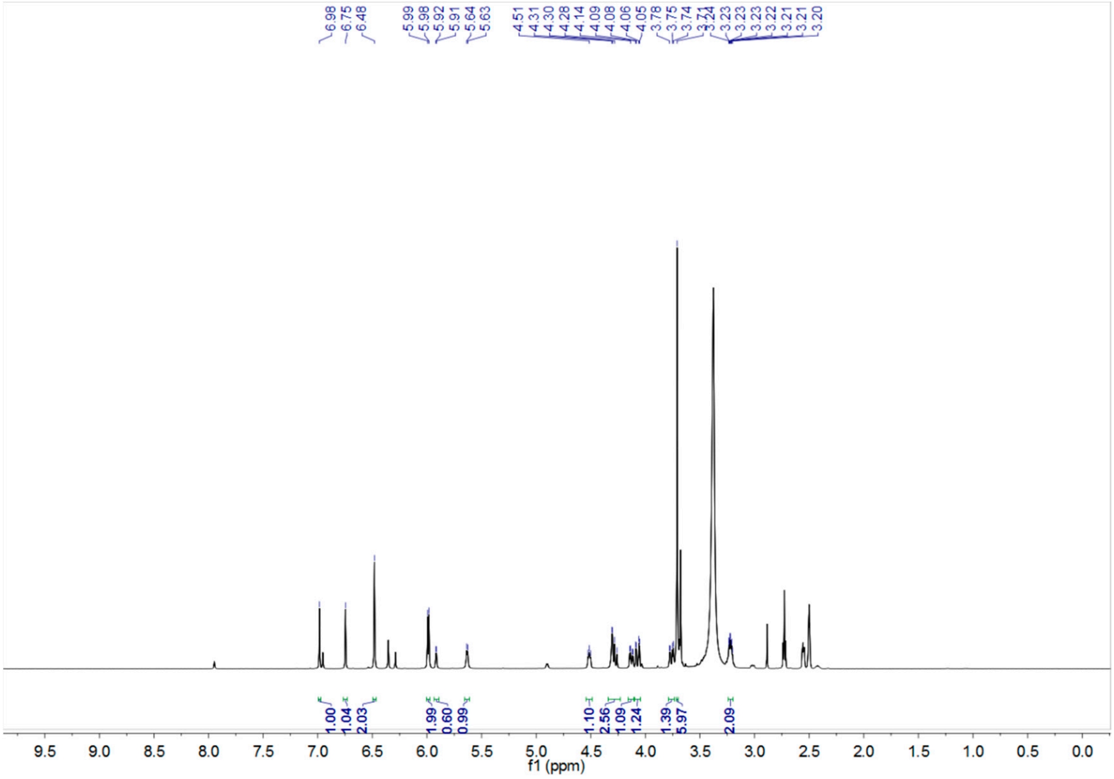

Figure S2. ^{1}H NMR spectrum (DMSO-d_6, 400 MHz) of compound $\mathbf{A_1}$.

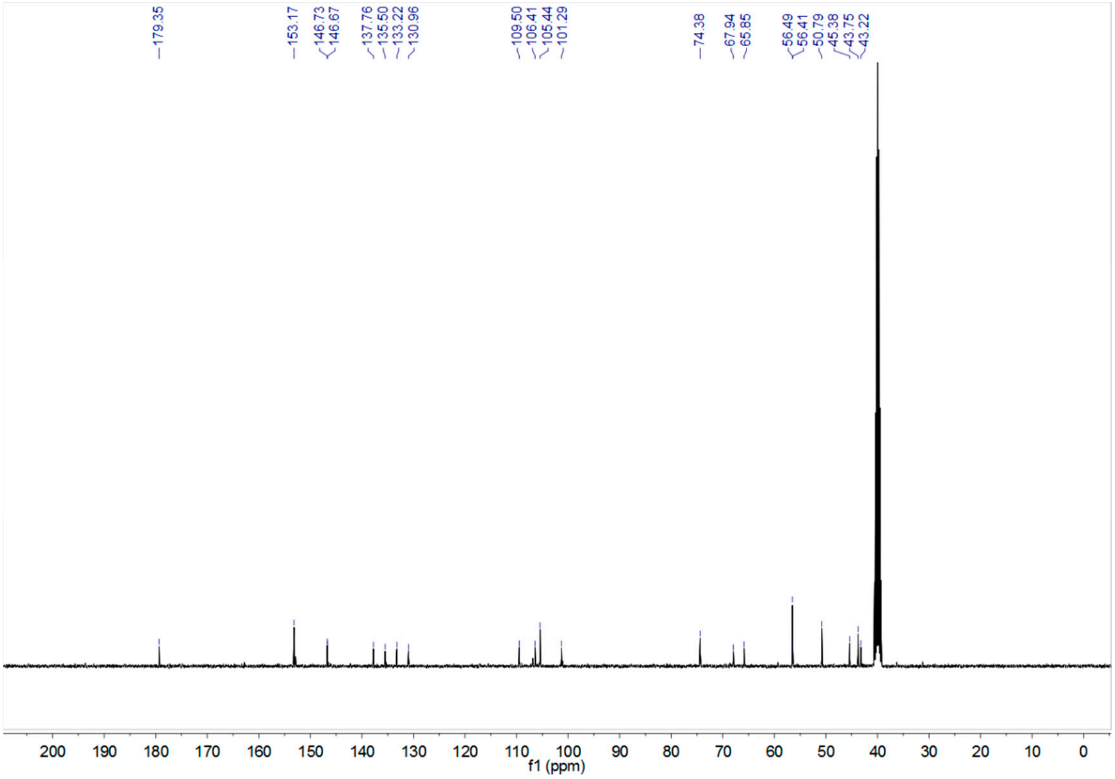

Figure S3. ^{13}C NMR spectrum (DMSO-d_6, 101 MHz) of compound **A$_1$**.

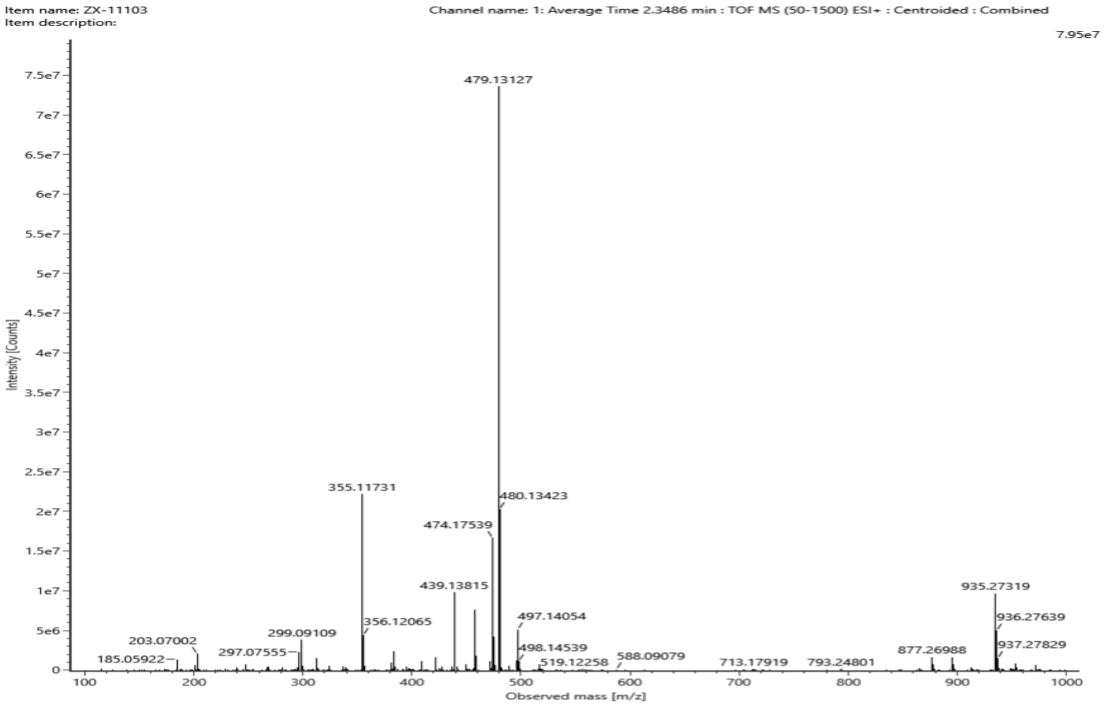

Figure S4. HRMS spectrum of compound **A$_1$**.

4-((5*R*,5a*R*,8a*R*,9*S*)-9-hydroxy-6-oxo-5,5a,6,8,8a,9-hexahydrofuro[3',4':6,7]naphtho[2,3-*d*][1,3]dioxol-5-yl)-2,6-dimethoxyphenyl 2-ethylbutanoate (B$_1$)

A white solid. ^{1}H NMR (400 MHz, $CDCl_3$) δ 6.86 (s, 1H, H-5), 6.50 (s, 1H, H-8), 6.27 (s, 2H, H-2′, 6′), 5.94 (dd, J = 1.2, 6.4 Hz, 2H, OCH_2O), 4.80 (d, J = 4.0 Hz, 1H, H-4), 4.59 (d, J = 5.2 Hz, 1H, H-1), 4.36-4.28 (m, 2H, H-11), 3.64 (s, 6H, 3′, 5′-OCH_3), 3.27 (dd, J = 4.8, 14.4 Hz, 1H, H-2), 2.82-2.73 (m, 1H, H-3), 2.48-2.41 (m. 1H, OCO<u>CH</u>(CH_2CH_3)$_2$), 1.79-1.68 (m, 2H, OCOCH(<u>CH_2</u>CH_3)$_2$), 1.64-1.54 (m, 2H, OCOCH(<u>CH_2</u>CH_3)$_2$), 0.99 (t, J = 7.2 Hz, 6H, OCOCH(CH_2<u>CH_3</u>)$_2$). ^{13}C NMR (101 MHz, $CDCl_3$) δ 175.44, 174.22, 151.52, 148.32, 147.36, 137.82, 132.17, 131.58, 127.83, 110.35, 109.29, 107.63, 101.50, 67.82, 66.42, 56.05, 50.55, 48.93, 43.97, 40.56, 25.33, 11.55. HRMS (ESI) calcd for $C_{27}H_{30}NaO_9$ ([M+Na]$^+$), 521.1788; found, 521.1786.

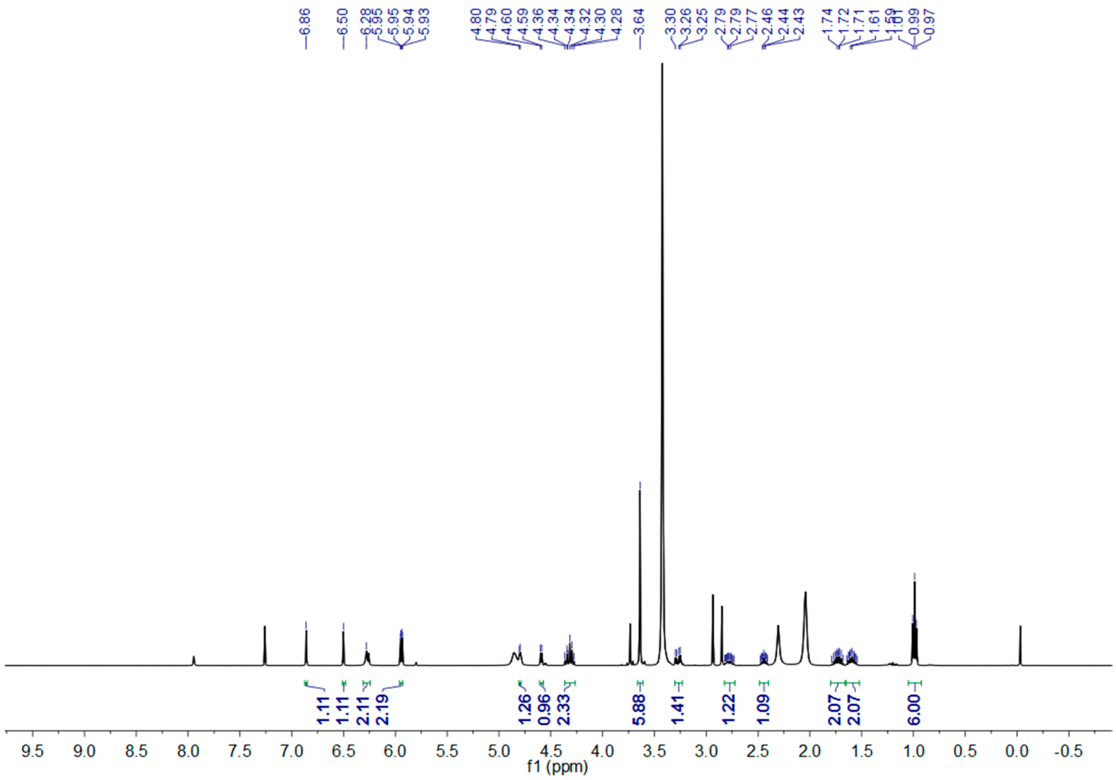

Figure S5. ^{1}H NMR spectrum ($CDCl_3$, 400 MHz) of compound **B_1**.

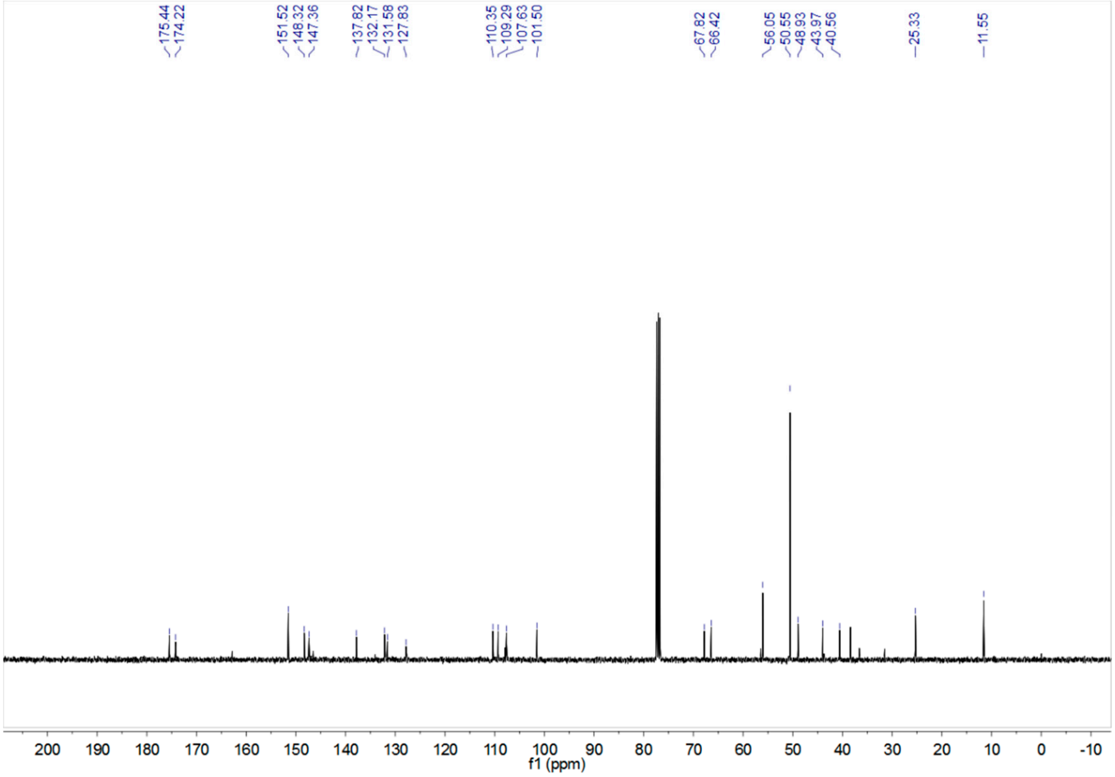

Figure S6. ^{13}C NMR spectrum ($CDCl_3$, 101 MHz) of compound **B$_1$**.

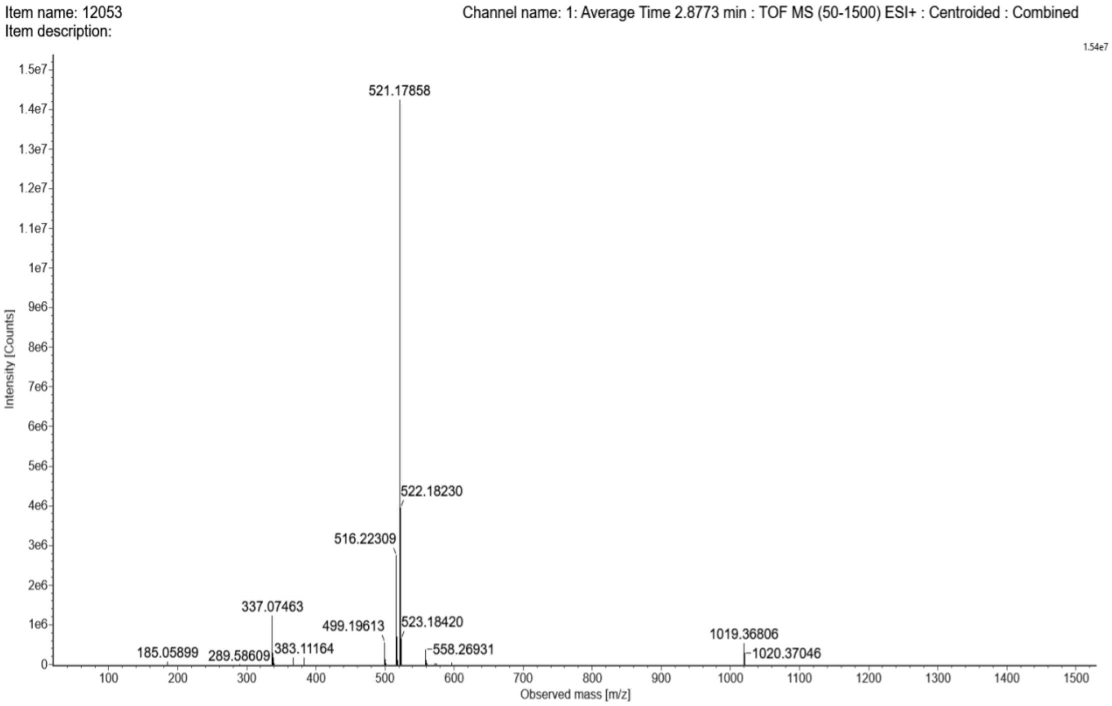

Figure S7. HRMS spectrum of compound **B$_1$**.

4-((5*R*,5a*R*,8a*R*,9*S*)-9-hydroxy-6-oxo-5,5a,6,8,8a,9-hexahydrofuro[3',4':6,7]naphtho[2,3-d][1,3]dioxol-5-yl)-2,6-dimethoxyphenyl 2-ethylhexanoate (B$_2$)

A white solid. ^{1}H NMR (400 MHz, $CDCl_3$) δ 6.86 (s, 1H, H-5), 6.54 (s, 1H, H-8), 6.30 (s, 2H, H-2′, 6′), 5.97 (dd, J = 1.2, 8.0 Hz, 2H, OCH_2O), 4.83 (s, 1H, H-4), 4.63 (d, J = 5.2 Hz, 1H, H-1), 4.38-4.28 (m, 2H, H-11), 3.66 (s, 6H, 3′, 5′-OCH_3), 3.27 (dd, J = 5.2, 14.0 Hz, 1H, H-2), 2.84-2.75 (m, 2H, H-3), 2.51-2.44 (m, 1H, OCOCH), 2.03 (s, 1H, $OCOCHCH_2CH_2CH_2CH_3$), 1.82-1.71 (m, 2H, $OCOCHCH_2CH_3$), 1.67-1.57 (m, 3H, $OCOCHCH_2CH_2CH_2CH_3$), 1.29-1.22 (m, 2H, $OCOCHCH_2CH_2CH_2CH_3$), 1.02 (t, J = 7.6 Hz, 6H, $OCOCHCH_2CH_3$ + $OCOCHCH_2CH_2CH_2CH_3$). ^{13}C NMR (101 MHz, $CDCl_3$) δ 175.00, 174.02, 151.59, 148.53, 147.54, 137.67, 131.97, 131.77, 127.95, 110.59, 109.04, 107.65, 101.61, 67.60, 66.71, 58.49, 56.10, 48.93, 43.99, 40.55, 38.26, 25.36, 25.35, 18.43, 11.60. HRMS (ESI) calcd for $C_{29}H_{34}NaO_9$ ($[M+Na]^+$), 549.2101; found, 549.2098.

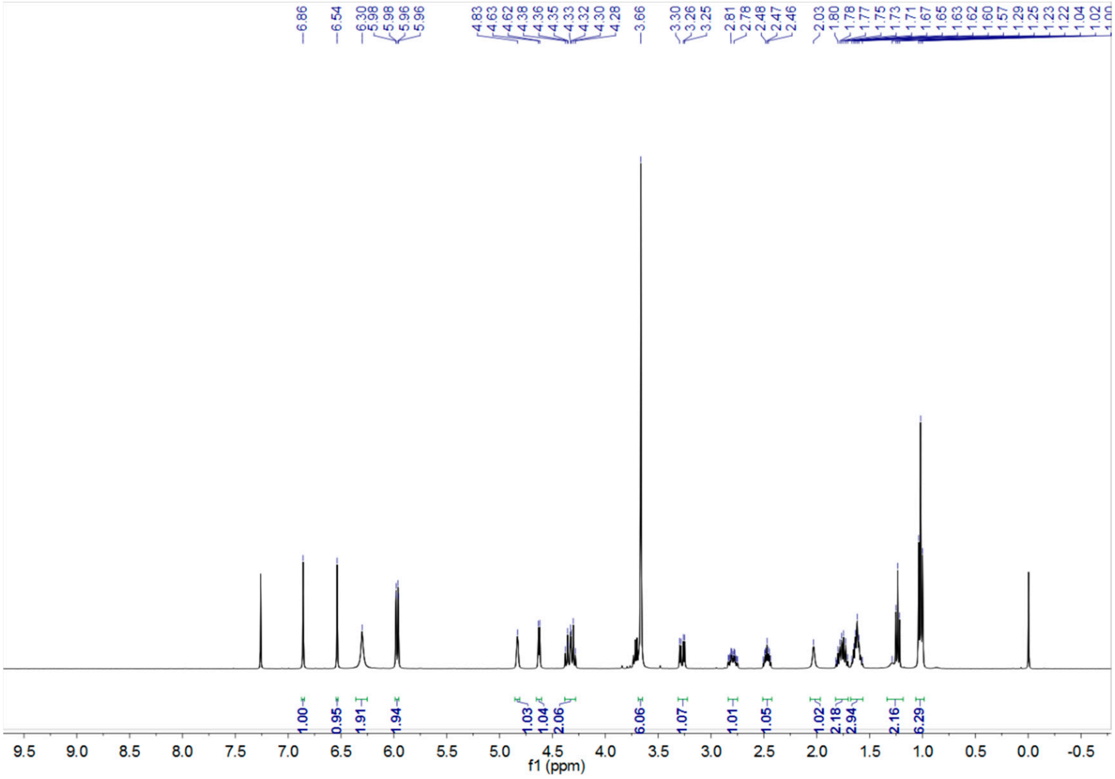

Figure S8. ^{1}H NMR spectrum ($CDCl_3$, 400 MHz) of compound **B_2**.

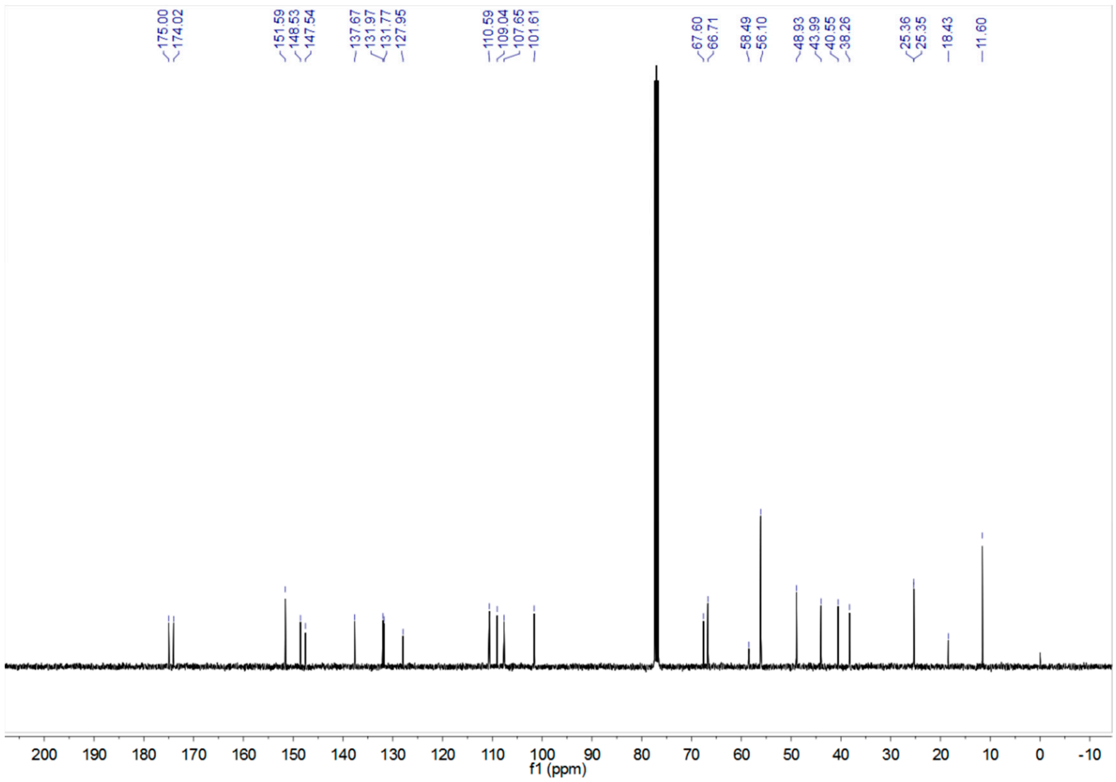

Figure S9. ^{13}C NMR spectrum ($CDCl_3$, 101 MHz) of compound **B_2**.

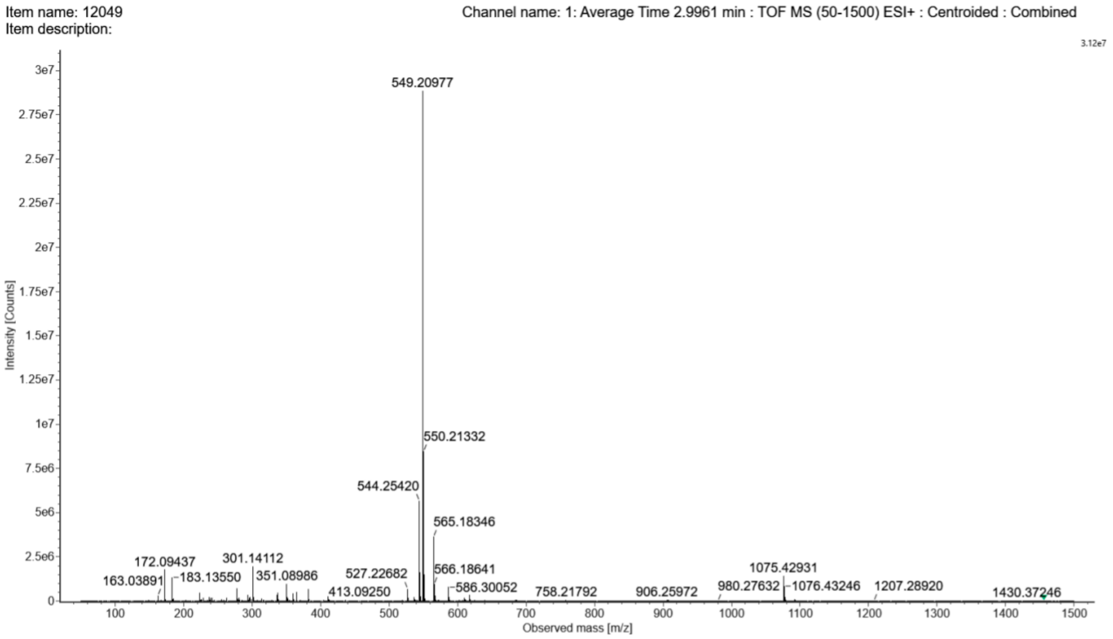

Figure S10. HRMS spectrum of compound **B_2**.

4-((5*R*,5a*R*,8a*R*,9*S*)-9-hydroxy-6-oxo-5,5a,6,8,8a,9-hexahydrofuro[3',4':6,7]naphtho[2,3-*d*][1,3]dioxol-5-yl)-2,6-dimethoxyphenyl 4-methylbenzenesulfonate (B_3)

A white solid. ^{1}H NMR (400 MHz, DMSO-d_6) δ 7.73 (d, J =8.0 Hz, 2H, Ar-H), 7.45 (d, J =8.0 Hz, 2H, Ar-H), 6.95 (s, 1H, H-5), 6.52 (s, 1H, H-8), 6.27 (s, 2H, H-2′, 6′), 6.00 (d, J = 2.0 Hz, 2H, OCH_2O), 5.47 (d, J = 6.0 Hz, 1H, H-4), 4.58 (d, J = 5.2 Hz, 1H, H-1), 4.38-4.33 (m, 1H, H-11), 4.22-4.17 (m, 1H, H-11), 3.45 (s, 6H, 3′, 5′-OCH_3), 3.30-3.22 (m, 1H, H-2), 2.81-2.75 (m, 1H, H-3), 2.42 (s, 3H, Ar-$\underline{CH_3}$). ^{13}C NMR (101 MHz, DMSO-d_6) δ 175.30, 152.26, 147.67, 146.91, 145.30, 140.81, 134.51, 133.86, 131.14, 129.96, 128.34, 126.52, 110.40, 109.78, 107.83, 101.64, 68.24, 65.35, 56.12, 46.17, 43.68, 38.77, 21.59. HRMS (ESI) calcd for $C_{28}H_{26}NaO_{10}S$ ([M+Na]$^+$), 577.1144; found, 577.1148.

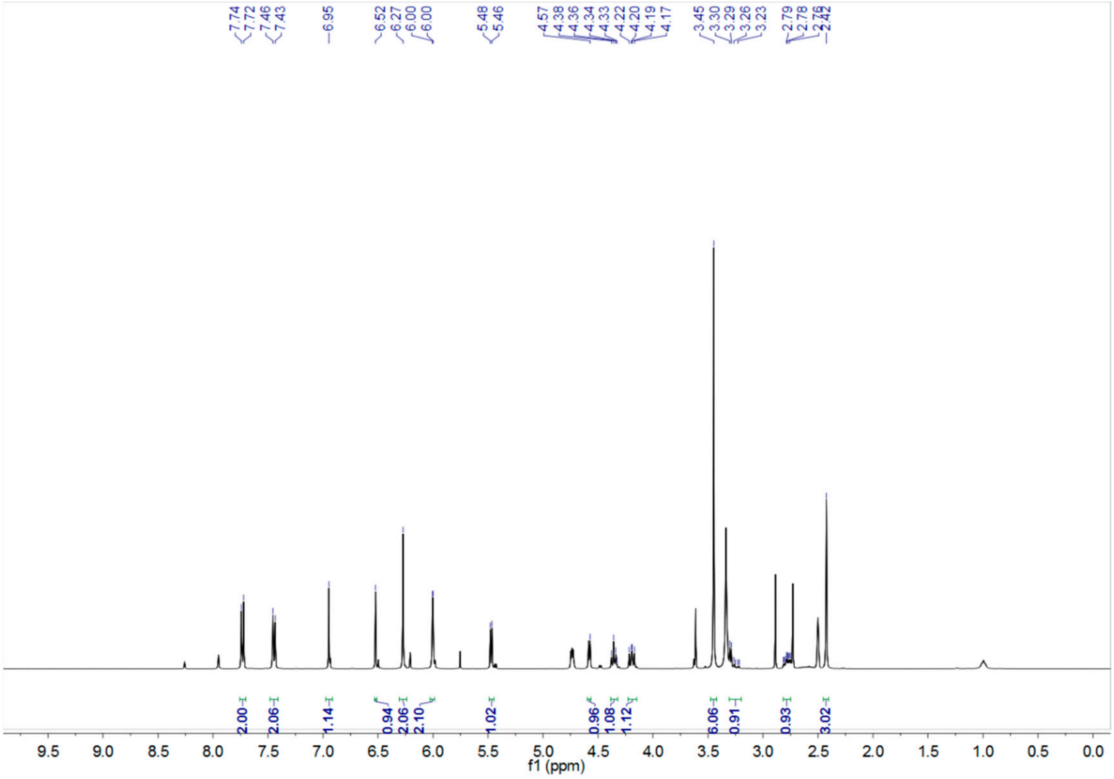

Figure S11. ^{1}H NMR spectrum (DMSO-d_6, 400 MHz) of compound **B$_3$**.

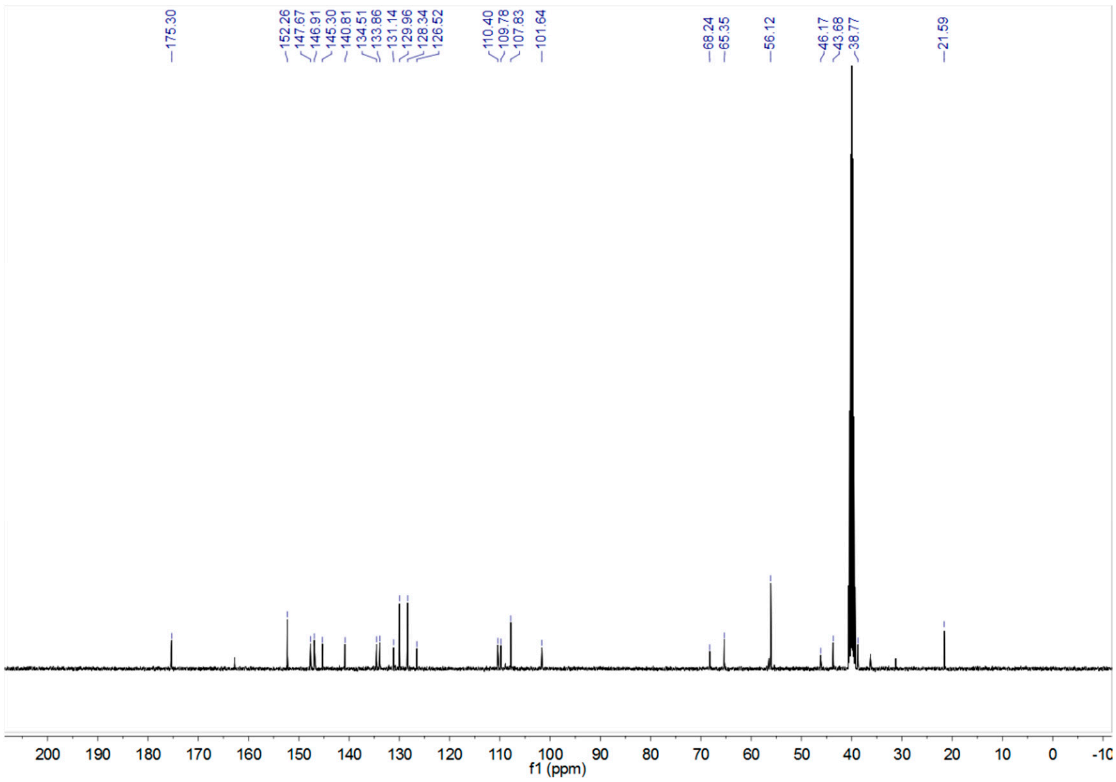

Figure S12. ^{13}C NMR spectrum (DMSO-d_6, 101 MHz) of compound **B3**.

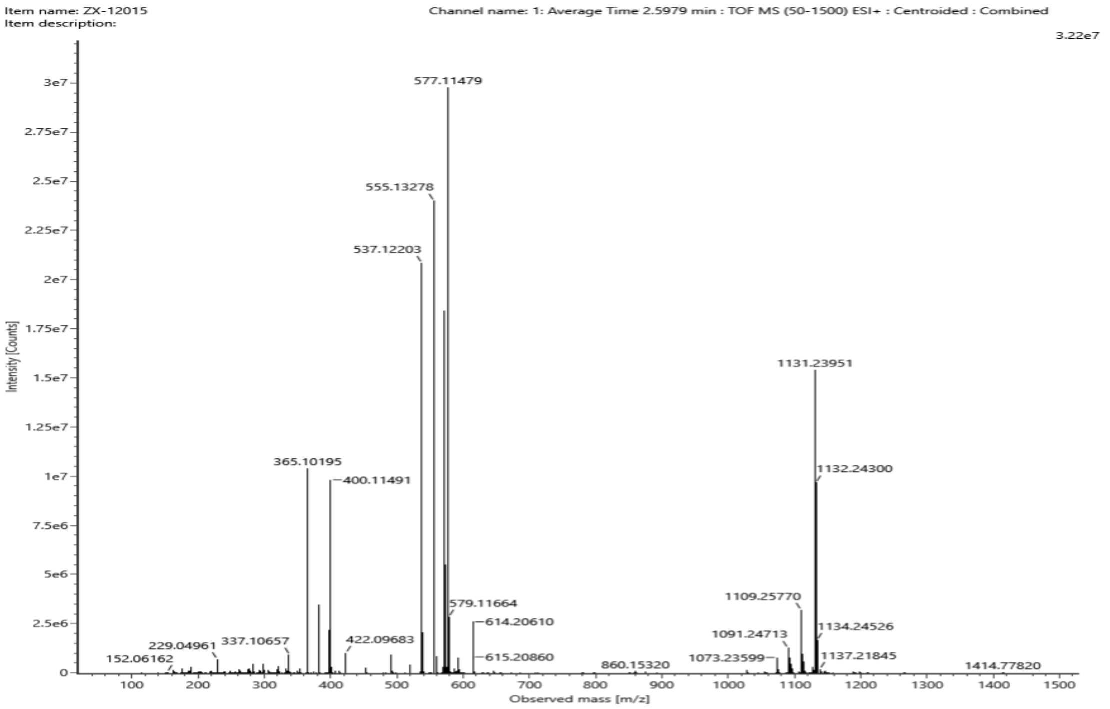

Figure S13. HRMS spectrum of compound **B3**.

Table S1 The docking scores of compounds with *Xoo*FtsZ

Entry	Total score	Crash	Polar	Cscore	Global Score
4'-demethylepipodophyllotoxin	5.92	-2.43	1.17	4	4
4'-demethylpodophyllotoxin	1.85	-2.34	0.97	1	1

References

1. Wang, P.Y.; Xiang, M.; Luo, M.; Liu, H.W.; Zhou, X.; Wu, Z.B.; Liu, L.W.; Li, Z.; Yang, S.; Novel piperazine-tailored ursolic acid hybrids as significant antibacterial agents targeting phytopathogens *Xanthomonas oryzae* pv. *oryzae* and *X. axonopodis* pv. *citri* probably directed by activation of apoptosis. *Pest Manag. Sci.* **2020**, *76*, 2746–2754.
2. Zhao, Y.L.; Huang, X.; Liu, L.W.; Wang, P.Y.; Long, Q.S.; Tao, Q.Q.; Li, Z.; Yang, S.; Identification of racemic and chiral carbazole derivatives containing an isopropanolamine linker as prospective surrogates against plant pathogenic bacteria: in vitro and in vivo assays and quantitative proteomics. *J. Agric. Food Chem.* **2019**, *67*, 7512–7525.
3. Liu, H.W.; Ji, Q.T.; Ren, G.G.; Wang, F.; Su, F.; Wang, P.Y.; Zhou, X.; Wu, Z.B.; Li, Z.; Yang, S.; Antibacterial functions and proposed modes of action of novel 1,2,3,4-tetrahydro-*β*-carboline derivatives that possess an attractive 1,3-diaminopropan-2-ol pattern against rice bacterial blight, kiwifruit bacterial canker, and citrus bacterial canker. *J. Agric. Food Chem.* **2020**, *68*, 12558–12568.
4. Tao, Q.Q.; Liu, L.W.; Wang, P.Y.; Long, Q.S.; Zhao, Y.L.; Jin, L.H.; Xu, W.M.; Chen, Y.; Li, Z.; Yang, S.; Synthesis and in vitro and in vivo biological activity evaluation and quantitative proteome profiling of oxadiazoles bearing flexible heterocyclic patterns. *J. Agric. Food Chem.* **2019**, *67*, 7626–7639.
5. Zeng, D.; Wang, M.W.; Xiang, M.; Liu, L.W.; Wang, P.Y.; Li, Z.; Yang, S.; Design, synthesis, and antimicrobial behavior of novel oxadiazoles containing various *N*-containing heterocyclic pendants. *Pest Manag. Sci.* **2020**, *76*, 2681–2692.
6. Xiang, M.; Song, Y.L.; Ji, J.; Zhou, X.; Liu, L.W.; Wang, P.Y.; Wu, Z.B.; Li, Z.; Yang, S.; Synthesis of novel 18*β*-glycyrrhetinic piperazine amides displaying significant in vitro and in vivo antibacterial activities against intractable plant bacterial diseases. *Pest Manag. Sci.* **2020**, *76*, 2959–2971.
7. Li, Y.; Zhou, X.; Wu, H.G.; Yu, Z.Z.; Li, H.; Yang, S.; Nanospheric heterogeneous acid-enabled direct upgrading of biomass feedstocks to novel benzimidazoles with potent antibacterial activities. *Ind. Crops Prod.* **2020**, *150*, 112406.
8. Zhou, X.; Feng, Y.M.; Qi, P.Y.; Shao, W.B.; Wu, Z.B.; Liu, L.W.; Wang, Y.; Ma, H.D.; Wang, P.Y.; Li, Z.; et al. Synthesis and docking study of *N*-(Cinnamoyl)-*N'*-(substituted) acryloyl hydrazide derivatives containing pyridinium moieties as a novel class of filamentous temperature-sensitive protein Z inhibitors against the intractable *Xanthomonas oryzae* pv. *oryzae* infections in rice. *J. Agric. Food Chem.* **2020**, *68*, 8132–8142.
9. Zhou, X.; Ye, Y.Q.; Liu, S.S.; Shao, W.B.; Li, L.W.; Yang, S.; Wu, Z.B.; Design, synthesis and anti-TMV activity of novel *α*-aminophosphonate derivatives containing a chalcone moiety that induce resistance against plant disease and target the TMV coat protein. *Pestic Biochem. Phys.* **2021**, *172*, 104749.
10. Singh, D.; Bhattacharya, A.; Rai, A.; Dhaked HPS, Awasthi, D.; Ojima, I.; Panda, D.; SB-RA-2001 inhibits bacterial proliferation by targeting FtsZ assembly. *Biochemistry* **2014**, *53*, 2979–2992.
11. Cai, S.Y.; Yuan, W.C.; Li, Y.; Huang, X.H.; Guo, Q.; Tang, Z.W.; Fang, Z.Y.; Lin, H.; Wong, W.L.; Wong, K.Y.; et al. Antibacterial activity of indolyl-quinolinium derivatives and study their mode of action. *Bioorg. Med. Chem.* **2019**, *27*, 1274–1282.
